# Tumor safety of biologic agents and targeted therapies in immune-mediated inflammatory arthritides

**DOI:** 10.3389/fimmu.2026.1827981

**Published:** 2026-06-04

**Authors:** Sheng-Guang Li, Yadan Zou, Ji Li, Lina Zhang, Jing Zhang, Ting Long, Ruohan Yu, Yanfeng Zhang

**Affiliations:** Department of Rheumatology and Immunology, Peking University International Hospital, Beijing, China

**Keywords:** axial spondyloarthritis, biologic DMARDs, cancer risk, immune-mediated inflammatory arthritides, JAK inhibitors, malignancy, psoriatic arthritis, rheumatoid arthritis

## Abstract

Immune-mediated inflammatory arthritides (IMIA), including rheumatoid arthritis, psoriatic arthritis, axial spondyloarthritis, and juvenile idiopathic arthritis, are chronic immune-driven disorders in which long-term malignancy safety has become a key determinant of therapeutic decision-making. Patients with IMIA are not oncologically neutral at baseline; persistent inflammatory burden, smoking exposure, age, and cumulative immunosuppressive treatment all modify cancer susceptibility. Against this background, biologic and targeted synthetic disease-modifying antirheumatic drugs may both reduce inflammation-associated oncogenic pressure and attenuate antitumor immune surveillance. In this narrative review, we synthesize current evidence on tumor safety across major biologic classes and Janus kinase inhibitors, with emphasis on data emerging through early 2026. Overall, most biologic therapies appear broadly reassuring with respect to overall malignancy, although non-melanoma skin cancer remains the most reproducible treatment-associated signal, particularly with tumor necrosis factor inhibitors and possibly abatacept. Rituximab retains a favorable profile in patients with prior lymphoproliferative disease, whereas IL-6 and IL-17/23 pathway inhibitors appear largely neutral or reassuring in currently available datasets. By contrast, JAK inhibitors require greater caution in risk-enriched rheumatoid arthritis populations, especially older patients, smokers, and those with prior malignancy or prolonged treatment exposure. Recent register-based studies have shown that overall cancer incidence with JAK inhibitors is comparable to that with TNF inhibitors, although lung and keratinocyte cancers occur more frequently in certain risk groups; accordingly, updated 2025 EULAR guidance recommends early initiation of targeted therapy after cancer remission and tailoring drug choice to prior cancer type and patient-specific factors. We further examine tumor-type-specific patterns, major modifiers of risk, and practical risk-stratified management strategies. The central clinical message is that malignancy safety in IMIA should be interpreted through an individualized framework that balances inflammatory control against oncologic vulnerability rather than through uniform class-based avoidance.

## 1 Introduction

Immune-mediated inflammatory arthritides (IMIA), encompassing rheumatoid arthritis (RA), psoriatic arthritis (PsA), axial spondyloarthritis (axSpA), and juvenile idiopathic arthritis (JIA), are chronic inflammatory disorders that share overlapping immune pathways but differ in tissue tropism, clinical phenotype, and therapeutic response (1–5). Across these diseases, persistent inflammation drives pain, functional impairment, structural damage, and systemic comorbidity, whereas the expanding use of biologic and targeted synthetic disease-modifying antirheumatic drugs (bDMARDs and tsDMARDs) has fundamentally shifted treatment goals from symptom control toward sustained remission, prevention of structural progression, and increasingly individualized therapy selection (2–5).

Malignancy safety has therefore become a major issue in long-term IMIA management. Importantly, these patients are not oncologically neutral before treatment begins. In RA, meta-analytic data indicate a modest increase in overall malignancy and a more pronounced excess of lymphoma and lung cancer compared with the general population (6). Registry evidence further suggests that lymphoma risk is strongly linked to cumulative inflammatory burden rather than to biologic exposure alone (7). This creates a biologically plausible duality: effective immunomodulation may reduce inflammation-driven oncogenic pressure, yet it may also attenuate antitumor immune surveillance and alter the host–tumor equilibrium (1, 6, 7).

This balance has become even more clinically consequential in the JAK inhibitor era. The ORAL Surveillance trial showed a higher incidence of malignancy excluding non-melanoma skin cancer with tofacitinib than with TNF inhibitors in risk-enriched RA (8), and a subsequent meta-analysis across immune-mediated diseases found a higher malignancy incidence with JAK inhibitors versus TNF inhibitors, but not versus placebo or methotrexate (9). At the same time, the 2024 EULAR points to consider emphasized that treatment decisions in patients with previous cancer should be individualized rather than governed by blanket therapeutic avoidance (10). Against this background, the present review synthesizes contemporary evidence on tumor safety across biologic therapies and JAK inhibitors in IMIA, with a focus on drug-class signals, tumor-specific patterns, risk modifiers, and practical management strategies.

## 2 Mechanistic basis linking chronic inflammation, immune modulation, and tumor risk

The relationship between IMIA, immunomodulatory therapy, and malignancy cannot be understood as a simple question of “drug toxicity.” Rather, cancer risk emerges from the interaction between a pre-existing inflammatory milieu, tissue-resident stromal remodeling, host antitumor immunity, and therapy-induced alterations in immune signaling. Chronic inflammatory states can provide several enabling conditions for carcinogenesis, including non-mutational epigenetic reprogramming, phenotypic plasticity, aberrant tissue repair, angiogenesis, and immune subversion (1, 11, 12). In IMIA, persistent activation of cytokine networks centered on TNF-α, IL-6, IL-17, and IL-23 sustains synovial and systemic inflammation, while also influencing oxidative stress, cell survival programs, and leukocyte trafficking. Accordingly, the net malignancy risk in treated IMIA reflects the balance between suppression of inflammation-driven oncogenic processes and attenuation of tumor immune surveillance.

### 2.1 Chronic inflammation as a pro-tumorigenic milieu

Chronic inflammation is now recognized as a biologically plausible driver of tumor initiation and progression. Persistent inflammatory signaling promotes the generation of reactive oxygen and nitrogen species (ROS/RNS), which can induce DNA damage, replication stress, and mutagenesis, while repeated tissue injury and repair favor stromal remodeling and clonal selection (11, 12). In cancer biology more broadly, these processes support hallmark capabilities such as survival signaling, angiogenesis, immune evasion, and altered differentiation states (11). Within IMIA, prolonged synovitis exposes resident stromal cells and infiltrating immune cells to a cytokine-rich environment dominated by TNF-α, IL-6, IL-17, and other mediators. IL-6 is particularly relevant because the IL-6/JAK/STAT3 axis links chronic inflammation to tumor cell proliferation, epithelial–mesenchymal transition, angiogenesis, and suppression of antitumor immunity (13, 14). IL-17 has similarly been implicated in inflammation-associated tumor progression through promotion of angiogenesis, myeloid recruitment, and cytokine amplification, although its role is context dependent and not uniformly pro-tumorigenic across tissues (15).

RA also offers a disease-specific model of how chronic inflammation can shape oncologic risk. Synovial fibroblasts in RA develop a quasi-transformed phenotype characterized by resistance to apoptosis, invasive behavior, epigenetic persistence, and matrix-destructive functions, leading some investigators to describe rheumatoid synovium as “tumor-like” from a stromal biology perspective (16). This does not mean that inflamed synovium is neoplastic, but it does underscore how chronic inflammatory remodeling may generate a microenvironment permissive to genomic instability, abnormal survival signaling, and tissue invasion (16). The most clinically established example of inflammation-associated malignancy in RA is lymphoma. In a classic case-control study, high cumulative inflammatory activity—not antirheumatic treatment itself—was strongly associated with increased lymphoma risk, especially diffuse large B-cell lymphoma (17). These observations provide an important conceptual anchor for interpreting the safety of targeted therapies: uncontrolled inflammation may itself be carcinogenic, particularly in relation to hematologic malignancy.

### 2.2 Immune surveillance and cancer immunoediting

Set against the pro-tumorigenic effects of chronic inflammation is the protective role of the immune system in tumor surveillance. The modern concept of cancer immunoediting describes three dynamic phases—elimination, equilibrium, and escape—through which immune cells can initially suppress emerging malignant clones, constrain their outgrowth, and ultimately shape the selection of variants capable of immune evasion (18). Cytotoxic T lymphocytes, natural killer (NK) cells, dendritic cells, and interferon-driven programs are central to the elimination phase, whereas chronic antigenic pressure and persistent inflammatory signaling can favor exhaustion, tolerance, and escape (18, 19). In this framework, tumor development is not merely the consequence of mutational events, but also of a progressive breakdown in immune recognition and containment.

Importantly, chronic inflammation does not simply coexist with immune surveillance; it can distort it. The tumor microenvironment often becomes enriched with regulatory T cells (Tregs), myeloid-derived suppressor cells (MDSCs), alternatively activated macrophages, and checkpoint-associated inhibitory signaling, all of which can reduce effective cytotoxic responses (12, 13). IL-6/STAT3 signaling is particularly notable in this regard because it promotes both tumor-cell intrinsic survival programs and extrinsic immune suppression. Likewise, persistent Th17-skewed inflammation may amplify tissue damage and myeloid recruitment while failing to generate durable tumor control (13, 15). Thus, chronic inflammation creates a paradox: it can increase the likelihood of malignant transformation and simultaneously undermine the host immune mechanisms that would otherwise eliminate transformed cells.

### 2.3 How targeted therapy may lower—or increase—tumor risk

This mechanistic duality explains why advanced therapies for IMIA can have bidirectional implications for cancer risk. On one hand, effective cytokine blockade may reduce inflammation-driven carcinogenesis by lowering oxidative stress, attenuating tissue injury–repair cycles, reducing stromal activation, and suppressing B-cell or myeloid activation associated with lymphomagenesis (12, 17). This may help explain why TNF inhibitors have generally not increased lymphoma incidence beyond the elevated background risk of RA and, in some settings, may normalize the risk associated with uncontrolled disease activity (7, 17). The same logic can be extended, at least conceptually, to other biologics: if a therapy meaningfully reduces chronic inflammatory burden without profoundly disabling antitumor immunity, its net effect on malignancy could be neutral or even protective in selected settings.

On the other hand, any therapy that modulates immune pathways involved in tumor surveillance may theoretically delay elimination of subclinical malignant clones. This concern is strongest when treatment affects antigen presentation, interferon signaling, NK-cell function, cytotoxic T-cell priming, or control of oncogenic viruses (18, 19). The mechanism likely differs across drug classes. Rituximab depletes B cells and may be neutral or even favorable in patients with prior B-cell lymphoproliferative disease, whereas abatacept, through T-cell costimulation blockade, could theoretically attenuate antitumor T-cell priming. Cytokine-specific biologics such as IL-6 or IL-17 inhibitors may exert narrower effects, whereas broader pathway inhibition may influence multiple immunologic checkpoints simultaneously (13, 15, 19). This is why the same clinical endpoint— “malignancy risk”—should not be interpreted as mechanistically uniform across all targeted therapies.

Recent advances in cancer immunotherapy further illustrate why tumor safety cannot be interpreted as a simple class-wide property. Immune checkpoint inhibitors that block CTLA-4 or PD-1/PD-L1 deliberately enhance antitumor T-cell activity (20), and this strategy has improved outcomes in immunogenic tumors such as melanoma and renal cell carcinoma (21, 22). This creates a conceptual mirror image of some anti-inflammatory strategies used in IMIA: ipilimumab blocks CTLA-4 signaling to release T-cell priming, whereas abatacept is a CTLA-4-Ig fusion protein that prevents CD28-mediated co-stimulation and dampens T-cell activation. Abatacept has also been used to reverse severe immune-related adverse events caused by checkpoint blockade (23), underscoring its capacity to suppress the same antitumor immune programs that oncology increasingly seeks to amplify. From this perspective, small observational signals for melanoma or renal/urological cancers under selected immunomodulators are biologically plausible, because these tumors are among the malignancies in which T-cell checkpoint pathways are therapeutically important; nevertheless, current IMIA data remain insufficient to establish drug-specific causality.

### 2.4 Why JAK inhibitors deserve separate mechanistic attention

JAK inhibitors warrant separate attention because the JAK/STAT pathway occupies a central position at the interface of inflammation, immune regulation, and cancer biology. JAK-dependent signaling transduces not only pathogenic cytokines involved in IMIA but also type I and type II interferons and several cytokines essential for cytotoxic lymphocyte and NK-cell biology (13, 19). From a mechanistic standpoint, this creates a genuine double-edged effect: suppressing JAK/STAT signaling may reduce inflammatory amplification and STAT3-driven tumor-promoting pathways, yet it may also blunt interferon-mediated immune surveillance and antitumor editing (13, 19). This broader immunologic reach distinguishes JAK inhibitors from more selective extracellular cytokine blockade and provides one plausible biological explanation for why their malignancy signal has differed from that of most biologics in risk-enriched RA populations (8, 9).

**Figure 1:**
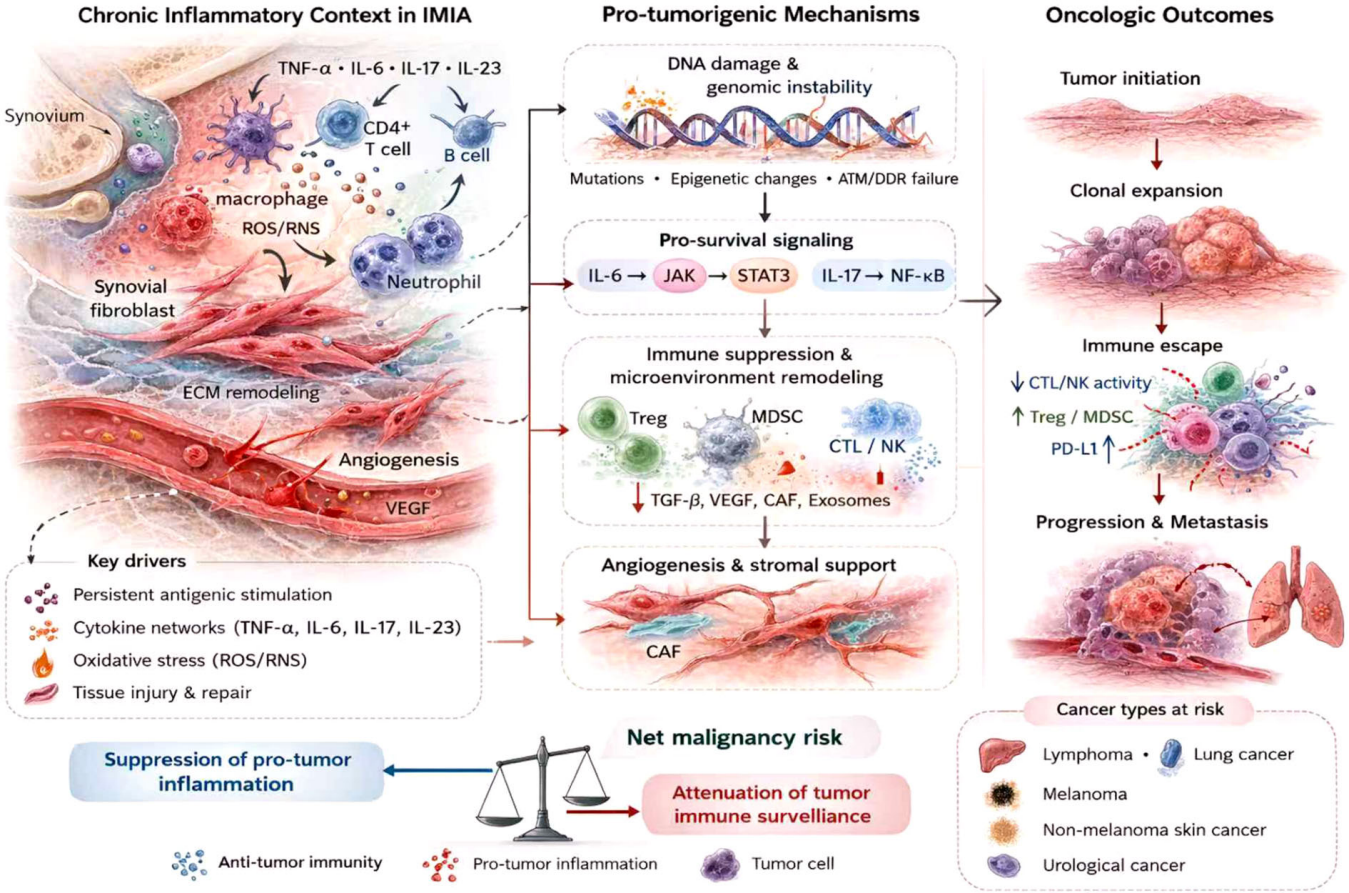
Inflammation–tumorigenesis interface in immune-mediated inflammatory arthritis. Chronic inflammation in immune-mediated inflammatory arthritis (IMIA) generates a complex tissue microenvironment that may promote tumorigenesis while simultaneously shaping anti- tumor immune responses. Persistent cytokine signaling involving tumor necrosis factor (TNF), interleukin-6 (IL-6), IL-17 and IL-23, together with oxidative stress and stromal remodeling, can induce DNA damage, genomic instability and epigenetic alterations. Pro-survival pathways such as IL-6–JAK–STAT3 and IL-17–NF-κB signaling further support tumor cell proliferation and survival. In parallel, expansion of immunosuppressive cell populations, including regulatory T cells (Tregs) and myeloid-derived suppressor cells (MDSCs), reduces cytotoxic T lymphocyte (CTL) and natural killer (NK) cell activity and facilitates immune escape. These processes contribute to tumor initiation, clonal expansion and metastatic progression. At the same time, effective control of inflammatory activity may reduce inflammation-driven oncogenic signaling. The overall malignancy risk therefore reflects a dynamic balance between suppression of pro-tumor inflammation and attenuation of tumor immune surveillance. IMIA, immune-mediated inflammatory arthritis; TNF, tumor necrosis factor; IL, interleukin; APC, antigen-presenting cell; ROS, reactive oxygen species; RNS, reactive nitrogen species; ECM, extracellular matrix; VEGF, vascular endothelial growth factor; CAF, cancer-associated fibroblast; DDR, DNA damage response; JAK, Janus kinase; STAT3, signal transducer and activator of transcription 3; NF-κB, nuclear factor-κB; Treg, regulatory T cell; MDSC, myeloid-derived suppressor cell; CTL, cytotoxic T lymphocyte; NK cell, natural killer cell; PD-L1, programmed death-ligand 1.

The clinical relevance of this mechanistic concern is supported by ORAL Surveillance, in which tofacitinib was associated with a higher incidence of malignancy than TNF inhibitors in older RA patients with cardiovascular risk factors (8), and by meta-analytic evidence showing a higher malignancy incidence with JAK inhibitors versus TNF inhibitors, but not versus placebo or methotrexate (9). These findings do not prove that all JAK inhibitors are intrinsically carcinogenic across all IMIA populations; rather, they suggest that broad cytokine signaling inhibition may become clinically relevant in patients whose baseline oncologic risk is already elevated by age, smoking, or cumulative inflammatory burden. In this sense, the JAK inhibitor experience reinforces the central theme of this section: tumor risk in IMIA is determined not by inflammation alone or therapy alone, but by the shifting balance between the two. This mechanistic interplay between chronic inflammation, immune surveillance and therapy-induced modulation is depicted in **Figure 1**.

## 3 Malignancy risk with biologic therapies in IMIA

Compared with the malignancy debate surrounding JAK inhibitors, the overall oncologic safety profile of biologic therapies in IMIA is substantially more reassuring. That reassurance, however, should not be oversimplified. Cancer outcomes differ by disease context, comparator choice, follow-up duration, prior immunosuppressive burden, and tumor subtype. In contemporary comparative cohorts, TNF inhibitors continue to function as the practical reference standard because they have the longest post-approval follow-up, whereas newer non-TNF biologics are often prescribed later in the disease course or in patients with more comorbidity, making confounding by indication a recurring interpretive challenge (24, 25). Thus, the most clinically useful question is not whether biologics are uniformly “safe” or “unsafe,” but whether any given biologic class confers a reproducible excess risk beyond the background malignancy burden intrinsic to IMIA and its host factors.

### 3.1 TNF inhibitors

TNF inhibitors remain the best-studied biologic class in inflammatory arthritis and, taken as a whole, do not appear to substantially increase the risk of overall malignancy. In a large U.S. claims-based cohort published in 2024, TNF inhibitors served as the reference category and were associated with lower incident cancer rates than rituximab and abatacept, while differences versus JAK inhibitors did not reach statistical significance within two years of follow-up (24). In a separate 2025 multi-database study using abatacept as the reference, TNF inhibitors showed no significant increase in overall malignancy risk (weighted HR 0.93, 95% CI 0.85–1.02), again supporting the view that TNF blockade is not linked to a broad excess cancer signal in routine RA care (25). These contemporary comparative studies are consistent with earlier registry-based data suggesting that TNF inhibitors, when evaluated against biologic-naive or conventionally treated RA populations, are largely neutral with respect to overall cancer incidence.

The most debated question for TNF inhibitors has been lymphoma. Because TNF-α participates in immune regulation and tumor surveillance, it was biologically plausible that prolonged TNF blockade might increase lymphoma risk. Yet observational evidence in RA has been consistently more reassuring than early theoretical concerns. In the British Society for Rheumatology Biologics Register, lymphoma incidence in TNF inhibitor–treated RA patients did not exceed that of biologic-naive RA patients after adjustment for disease-related factors (7). More recently, a 2024 systematic review and meta-analysis found no statistically significant increase in lymphoma risk with anti-TNF therapy compared with conventional treatment, although heterogeneity across observational studies remained high and the confidence intervals were wide (26). Taken together, these data suggest that lymphoma risk in TNF-exposed RA is driven more by cumulative inflammatory burden than by TNF inhibition itself, an interpretation that aligns with the mechanistic framework discussed in the previous section (7, 26).

The most consistent malignancy signal associated with TNF inhibitors concerns skin cancer. In the Swedish ARTIS program, TNF inhibitor exposure in RA was associated with a modest increase in invasive melanoma compared with biologic-naive RA (HR 1.5, 95% CI 1.0–2.2), although a later collaborative analysis across 11 European biologic registers did not confirm a statistically significant overall melanoma excess (IRR 1.1, 95% CI 0.8–1.6) (27, 28). This discrepancy likely reflects the difficulty of studying rare cancer outcomes across heterogeneous populations and underscores the need to interpret melanoma risk cautiously rather than dogmatically. By contrast, the evidence for non-melanoma skin cancer (NMSC), especially squamous cell carcinoma, is more coherent. In Sweden, TNF inhibitors were associated with a further increase in squamous cell carcinoma compared with biologic-naive RA (HR 1.30, 95% CI 1.10–1.55), whereas the excess for basal cell carcinoma was much less convincing (29). A 2020 meta-analysis similarly found that RA patients receiving TNF antagonists had an increased risk of NMSC, particularly squamous cell carcinoma (30). More recently, a 2025 meta-analysis in psoriasis and psoriatic arthritis found no increase in overall cancers excluding NMSC, melanoma, lymphoma, prostate cancer, or breast cancer with long-term TNF inhibitor therapy, but did report elevated NMSC risk in PsA and a significant increase in squamous cell carcinoma among psoriasis patients (31). These convergent findings make skin surveillance—not generalized cancer alarmism—the most rational safety response to long-term TNF inhibitor therapy.

Overall, TNF inhibitors should still be viewed as having a broadly reassuring malignancy profile in IMIA. Their main caveat is not a clear signal for lymphoma or solid tumor excess, but rather a reproducible association with NMSC and a possible modest melanoma signal in selected datasets. This distinction matters clinically: it supports continued use of TNF inhibitors in many patients, including some with prior solid tumors, while reinforcing the need for dermatologic vigilance in those with prior actinic damage, previous skin cancer, or substantial ultraviolet exposure (7, 24–31).

### 3.2 IL-6 inhibitors

The malignancy profile of IL-6 pathway inhibition appears neutral to reassuring. This is mechanistically plausible: although IL-6 supports inflammation and can promote tumor progression through STAT3-mediated pathways, selective IL-6 blockade may reduce inflammation-associated oncogenic pressure without producing the broader immune perturbation seen with JAK inhibition. The clinical evidence is consistent with that expectation. In a large U.S. multi-database cohort study, tocilizumab initiators had no greater risk of malignancy excluding NMSC than TNF inhibitor initiators (pooled HR 0.98, 95% CI 0.80–1.19), and risk was similarly comparable versus abatacept (32). In the 2025 Danish nationwide cohort, tocilizumab/sarilumab-treated RA patients likewise showed no statistically significant increase in overall cancer risk compared with TNF inhibitor-treated or biologic-naive RA populations (33). A 2020 meta-analysis of observational studies reached the same general conclusion, reporting no increase in overall malignancy with tocilizumab relative to csDMARDs or TNF inhibitors (34).

Recent comparative analyses have even raised the possibility that IL-6 inhibitors may compare favorably with some other biologics in selected cohorts. In the 2025 U.S. multi-database real-world study, IL-6 inhibitor initiators had a lower observed malignancy risk than abatacept initiators (weighted HR 0.73, 95% CI 0.60–0.88), although the authors explicitly cautioned that residual confounding and channeling bias may explain part of that apparent advantage (25). This is an important reminder that “reassuring” does not necessarily mean “protective.” At present, there is no robust evidence that IL-6 blockade reduces malignancy risk below baseline, only that it has not shown a consistent excess signal. Thus, IL-6 inhibitors can be considered among the more oncologically reassuring non-TNF biologics, particularly when JAK inhibitors are undesirable and TNF inhibitors are contraindicated or ineffective (25, 32–34).

### 3.3 IL-17 and IL-23 pathway inhibitors

For IL-17 and IL-23 pathway inhibitors, the cumulative evidence is also reassuring, although much of it derives from psoriasis, PsA, and axial spondyloarthritis trial programs rather than from long-term IMIA-specific registries comparable to those available for RA. Secukinumab has the most mature dataset. In an integrated analysis of 49 clinical trials plus post-marketing surveillance across psoriasis, PsA, and ankylosing spondylitis, the exposure-adjusted incidence rate of malignancy was 0.85 per 100 patient-years over five years (95% CI 0.74–0.98), with a standardized incidence ratio of 0.99 (95% CI 0.82–1.19) relative to the SEER reference population (35). An updated pooled safety analysis likewise found malignancy rates ≤1 per 100 patient-years across indications, without evidence of increasing incidence over time (36). These data do not suggest a class-specific oncologic penalty for IL-17A inhibition.

Broader synthesis of the IL-17/23 field points in the same direction. A systematic review and meta-analysis of adult patients with psoriasis and psoriatic arthritis found no short-term increase in malignancy with IL-17 or IL-23 antagonists and low long-term exposure-adjusted incidence rates for malignancy excluding NMSC (0.24 per 100 patient-years for IL-17 inhibitors and 0.43 per 100 patient-years for IL-23 inhibitors) (37). For NMSC, the long-term exposure-adjusted rates were also low (0.47 and 0.40 per 100 patient-years, respectively), with no evidence of progressive increase with longer follow-up (37). While these data are not directly equivalent to disease-specific cancer registries, they are highly relevant because they encompass the biologic mechanisms and clinical populations in which these agents are now commonly used.

The 2024–2025 ixekizumab literature further strengthens this reassuring picture. In a pooled analysis of 25 randomized clinical trials across psoriasis, PsA, and axial spondyloarthritis, malignant neoplasms remained infrequent and stable over time, with incidence rates of 0.7 per 100 patient-years in PsA and 0.4 per 100 patient-years in axSpA; standardized incidence ratios were near or below 1 across indications (38). A 2025 rheumatology-focused systematic review and meta-analysis found low malignancy risk in PsA and axial spondyloarthropathy, with pooled incidence rates of 0.31 per 100 patient-years at week 52 and 0.58 per 100 patient-years at 156 weeks; the authors concluded that ixekizumab appears to confer a low malignancy risk in rheumatologic indications, while appropriately noting that NMSC still warrants attention (39).

For IL-23-directed therapy, the long-term safety profile is similarly reassuring. In a 2024 comprehensive analysis of risankizumab across psoriatic disease trials, the rates of malignant tumors excluding NMSC and of NMSC were both 0.5–0.6 events per 100 patient-years in psoriasis and PsA populations, with no additional safety concerns emerging over time (40). Earlier integrated safety data for ustekinumab, including patients with psoriasis and PsA, also showed malignancies to be rare and comparable to placebo during controlled follow-up (0.4 vs 0.2 per 100 patient-years) (41). Collectively, these data support the view that IL-17/23 pathway inhibitors have a favorable oncologic profile, with the caveat that ultra-long-term comparative data in IMIA remain less mature than for TNF inhibitors. In practice, this makes them attractive options—especially in psoriatic disease phenotypes—when cancer risk is a major treatment consideration.

### 3.4 Abatacept

Abatacept deserves separate treatment because its mechanism—T-cell costimulation blockade—raises a distinct immunologic question: whether reduced T-cell priming could impair tumor immune surveillance more than cytokine-selective biologics do. The clinical evidence remains mixed. Observational studies have consistently identified a small but persistent signal for malignancy, especially NMSC. In a population-based comparative cohort, abatacept initiation as the first biologic in RA was associated with a modest increase in overall cancer incidence compared with other bDMARDs (adjusted HR 1.17, 95% CI 1.06–1.30), with the clearest site-specific signal being NMSC (adjusted HR 1.20, 95% CI 1.03–1.39) (42). More recently, a 2024 comprehensive evaluation of abatacept and NMSC found no significant difference versus placebo in randomized trials, but observational data suggested a higher NMSC risk compared with csDMARDs (pooled RR 1.84, 95% CI 1.00–3.37) and a borderline signal versus other b/tsDMARDs (RR 1.11, 95% CI 0.98–1.26) (43).

The 2025 meta-analysis across disease indications refined this picture. In pooled RCT and long-term extension data, abatacept was not associated with higher malignancy incidence than placebo or TNF inhibitors. By contrast, observational studies showed a higher incidence relative to other b/tsDMARDs (IRR 1.21, 95% CI 1.15–1.28) but not compared with csDMARDs (44). This discrepancy is highly informative: it suggests that at least part of the observational signal may reflect channeling bias rather than a strong intrinsic carcinogenic effect. Indeed, in contemporary routine-care datasets abatacept is often used in older, more treatment-experienced, or comorbidity-burdened patients, all of whom may carry higher baseline cancer risk. The 2024 U.S. claims study, for example, found higher incident cancer with abatacept than with TNF inhibitors over the first two years, but the authors explicitly interpreted this finding in light of likely confounding by indication (24). Thus, the most balanced interpretation is that abatacept has a plausible but still not fully resolved small malignancy signal—most clearly for NMSC—and deserves caution and dermatologic vigilance rather than blanket avoidance.

### 3.5 Rituximab

Rituximab is unique among IMIA biologics because it is also an established antineoplastic therapy for B-cell lymphomas. For that reason, its malignancy profile must be interpreted in the context of both mechanism and clinical use patterns. Long-term trial and post-marketing data have been reassuring. In analyses of 409,706 RA patients from the global company safety database and long-term clinical trial programs, no evidence of increased malignancy of any organ-specific type was found following rituximab exposure; the malignancy rate in RA trials was 7.4 per 1000 patient-years, with no increase over time or with additional rituximab courses (45). The final 11-year report of the global RA clinical trial program similarly found no increase in malignancy with prolonged exposure (46). These datasets strongly support the view that rituximab itself is not a driver of excess cancer risk in RA.

Recent real-world comparative studies appear more complicated, but mainly because of confounding. In the 2025 Danish cohort, rituximab was not associated with increased overall cancer risk compared with TNF inhibitors or biologic-naive RA (33). Yet in the 2024 U.S. claims study, rituximab initiators had a higher risk of incident cancer than TNF inhibitor initiators (HR 1.91, 95% CI 1.17–3.14), and in the 2025 multi-database study rituximab showed a higher malignancy risk than abatacept (weighted HR 1.30, 95% CI 1.14–1.49) (24, 25). These differences are very unlikely to be explained by a true carcinogenic effect of rituximab alone. Rituximab is frequently prescribed to older patients, patients with more refractory RA, and notably to patients with prior lymphoproliferative malignancy or prior biologic failure—precisely the populations expected to carry higher background oncologic risk. That interpretation is reinforced by the BIOBADASER registry, which found no adjusted increase in incident cancer with anti-CD20 therapy compared with TNF inhibitors among patients with prior malignancy (47). In short, rituximab remains one of the most reassuring biologic options from a mechanistic and long-term safety standpoint, while observational “excess risk” signals should be viewed primarily through the lens of channeling bias.

### 3.6 Interim synthesis

Taken together, the biologic classes used in IMIA do not show a uniform malignancy pattern. TNF inhibitors have the richest long-term evidence base and remain broadly reassuring with respect to overall malignancy, although NMSC and possibly melanoma require attention (24–31). IL-6 inhibitors also appear largely neutral and may be among the more oncologically reassuring non-TNF options (25, 32–34). IL-17 and IL-23 pathway inhibitors have shown low absolute malignancy rates and no convincing emerging cancer signal in long-term pooled programs, though disease-specific real-world evidence is still developing (35–41). Abatacept is the one biologic for which a small observational malignancy signal—particularly for NMSC—has remained relatively persistent, even though randomized and long-term extension datasets have not confirmed a clear excess (42–44). Rituximab, by contrast, appears oncologically reassuring when long-term data and indication bias are considered, and it retains a special place in patients with prior lymphoproliferative disease (33, 45–47). This class-specific heterogeneity is exactly why biologic-associated malignancy risk should be interpreted in a stratified rather than generalized manner, and it provides the essential backdrop for the next section, which addresses the more contested safety profile of JAK inhibitors. A summary of the overall and site-specific malignancy profiles for each biologic class is provided in **Table 1**.

**Table 1:** Drug-class-specific malignancy profile in IMIA: overall signal, dominant concerns, and practical interpretation.

Drug class	Main malignancy signal	Practical implication
TNF inhibitors	Overall cancer risk largely neutral; NMSC is the most consistent signal, with possible melanoma signal in selected cohorts.	Useful long-term comparator; maintain skin surveillance, especially with prior actinic damage or skin cancer.
IL-6 inhibitors	Neutral or reassuring; no reproducible site-specific malignancy signal.	Reasonable option when JAK inhibitors are undesirable or when TNF inhibitors are unsuitable.
IL-17/23 inhibitors	Low rates in long-term trial programs; comparative IMIA-specific registry data remain less mature.	Attractive in psoriatic phenotypes; continue routine malignancy surveillance.
Abatacept	Small observational signal, mainly for NMSC; randomized and extension data are not definitive.	Use thoughtfully in cancer-prone patients; consider dermatologic vigilance.
Rituximab	Reassuring overall; no convincing intrinsic carcinogenic signal.	Favored when prior lymphoma or lymphoproliferative concern is relevant.
JAK inhibitors	Greater concern in risk-enriched RA; lung cancer and NMSC are the most clinically relevant signals.	Use selectively in older patients, smokers, and those with prior malignancy after explicit benefit-risk discussion.

This table provides a concise drug-class summary of the dominant malignancy signals and practical clinical implications for biologic and targeted therapies used in IMIA.

IMIA, immune-mediated inflammatory arthritis; TNF, tumor necrosis factor; TNFi, tumor necrosis factor inhibitor; IL, interleukin; JAKi, Janus kinase inhibitor; NMSC, non-melanoma skin cancer; RA, rheumatoid arthritis.

## 4 Malignancy risk with JAK inhibitors

Among advanced therapies for IMIA, JAK inhibitors occupy a distinct position in malignancy discussions because their mechanism is broader than that of most biologics and their safety profile has been shaped by both randomized trial evidence and subsequent regulatory scrutiny. The strongest signal arose in rheumatoid arthritis rather than across IMIA as a whole, and it is therefore important to distinguish between class-wide caution and molecule-, disease-, and patient-specific risk. Overall, the available evidence suggests that malignancy concerns with JAK inhibitors are most relevant in risk-enriched RA populations, particularly for tofacitinib, whereas the magnitude of risk is less clear in lower-risk patients and in non-RA indications. This nuance is essential when interpreting the class as a whole (8, 9, 24, 48).

### 4.1 Pivotal randomized evidence: ORAL Surveillance and *post hoc* analyses

The modern debate on JAK inhibitors and malignancy is anchored in the ORAL Surveillance trial, a post-marketing randomized safety study that enrolled RA patients aged ≥50 years with at least one additional cardiovascular risk factor and compared tofacitinib (5 mg or 10 mg twice daily) with TNF inhibitors on background methotrexate (8). In the primary report, cancers excluding non-melanoma skin cancer (NMSC) occurred in 4.2% of patients receiving combined-dose tofacitinib versus 2.9% of those receiving TNF inhibitors, corresponding to a hazard ratio of 1.48 (95% CI 1.04–2.09) (8). A dedicated malignancy analysis subsequently showed that the incidence curves for malignancy excluding NMSC were similar up to approximately 18 months and then diverged thereafter; from month 18 onward, the hazard ratio for combined-dose tofacitinib versus TNF inhibitors was 1.93 (95% CI 1.22–3.06) (49). In the same analysis, lung cancer was the most frequent malignancy subtype in the tofacitinib groups, and NMSC incidence was also higher with tofacitinib than with TNF inhibitors (49). These findings remain the most persuasive randomized evidence that JAK inhibition—at least with tofacitinib in a high-risk RA population—may confer a malignancy disadvantage relative to TNF blockade.

Subsequent *post hoc* analyses have helped refine who appears to be driving this excess risk. In a clinically important stratified analysis, patients aged ≥65 years or ever-smokers constituted a “high-risk” subgroup in whom tofacitinib was associated with higher risks of malignancy excluding NMSC, major adverse cardiovascular events, venous thromboembolism, and death compared with TNF inhibitors; hazard ratios across these outcomes ranged from 1.41 to 5.19 (50). By contrast, among patients aged <65 years who had never smoked, there was no detectable increase in malignancy risk with tofacitinib versus TNF inhibitors over up to six years of follow-up, and absolute risk remained low (50). These findings do not eliminate concern, but they strongly suggest that the malignancy signal is not biologically or clinically uniform across all JAK inhibitor recipients. Rather, it appears to emerge most clearly in patients whose baseline oncologic and cardiometabolic risk is already elevated by age, smoking, and shared host susceptibility factors (49, 50).

### 4.2 Real-world and registry evidence: replication, attenuation, and heterogeneity

The critical question after ORAL Surveillance was whether the same signal would persist in routine care. The answer has been mixed. In the U.S. STAR-RA study, which compared tofacitinib initiators with TNF inhibitor initiators in both a broad real-world RA cohort and an ORAL Surveillance–like trial-emulation subset, the pooled weighted hazard ratio for malignancy excluding NMSC was 1.01 (95% CI 0.83–1.22) in routine care and 1.17 (95% CI 0.85–1.62) in the trial-duplicate cohort (51). These estimates were directionally consistent with ORAL Surveillance but notably attenuated and statistically non-significant, suggesting that the trial signal may not translate uniformly to all real-world RA patients (51). However, STAR-RA also emphasized that its results could not exclude a risk increase emerging with longer exposure, which is particularly relevant given the delayed separation of risk curves seen in ORAL Surveillance (49, 51).

A 2024 U.S. claims-based cohort added further perspective. In that study, JAK inhibitor initiators had a higher incident cancer risk than TNF inhibitor initiators within the first two years of follow-up, but the adjusted estimate was modest (HR 1.36, 95% CI 0.94–1.96) and did not reach statistical significance (24). The authors explicitly highlighted likely channeling bias, since patients receiving JAK inhibitors may have had higher baseline disease burden or a different comorbidity profile than TNF inhibitor users (24). Thus, the U.S. observational experience neither fully replicates nor refutes ORAL Surveillance; instead, it supports the idea that risk may be context dependent and sensitive to baseline patient characteristics, comparator choice, and follow-up duration.

European registry studies have provided some of the most informative post-trial data. In the Swedish ARTIS-linked cohort, RA patients initiating JAK inhibitors had no increase in overall cancer excluding NMSC compared with TNF inhibitor initiators (HR 0.94, 95% CI 0.65–1.38), but did show an increased NMSC risk (HR 1.39, 95% CI 1.01–1.91), with a stronger signal after at least two years of treatment (HR 2.12, 95% CI 1.15–3.89) (52). In the German RABBIT register, by contrast, JAK inhibitor–treated patients had a modestly increased risk of incident malignancy excluding NMSC relative to bDMARD-treated patients (adjusted HR 1.40, 95% CI 1.09–1.80; incidence rate 11.6 vs 8.9 per 1000 patient-years), and this difference was observed mainly in treatment episodes lasting longer than 16 months (53). RABBIT also found that the relative increase appeared more pronounced in patients aged ≥60 years, those with ≥3 prior csDMARD exposures, and those with high disease activity (53). These two registry programs are not contradictory so much as complementary: ARTIS suggests that NMSC may be the most reproducible early signal, whereas RABBIT suggests that overall cancer risk may rise modestly in more treatment-exposed or higher-risk RA populations if follow-up is long enough (52, 53).

A 2025 case–control study using the U.S. SEER–Medicare database compared 12,463 cancer cases and 38,345 cancer-free controls aged ≥65 years. Exposure to JAK inhibitors over a median of 1.8 years was not associated with an increase in overall cancer risk compared with TNF inhibitors or other biologics (adjusted odds ratio 1.04, 95% CI 0.87–1.26); however, JAK inhibitor use was linked to a higher risk of lung cancer, particularly in males and with treatment durations exceeding two years (1.40, 1.06–1.87) (54).

An international register-based analysis presented at EULAR 2025 further reported that while total cancer incidence did not differ between JAK inhibitors and TNF inhibitors, keratinocyte cancers occurred more frequently with JAK inhibitors (hazard ratio 1.72 vs TNF inhibitors) and the risk of a second keratinocyte cancer among those with prior lesions was markedly higher (2.76 vs TNF inhibitors) (55). These findings underscore the importance of systematic skin surveillance in patients receiving JAK inhibitors.

### 4.3 Agent-specific and long-term safety data

Because most of the class-level concern comes from tofacitinib, it is important to examine whether long-term integrated safety data across individual agents are compatible with a generalized carcinogenic effect. For tofacitinib, the largest integrated analysis of the RA clinical development program (up to 9.5 years of exposure) reported incidence rates of 0.8 per 100 patient-years for malignancy excluding NMSC, 0.6 for NMSC, and 0.1 for lymphoma, with rates remaining generally stable over time; the age- and sex-adjusted standardized incidence ratio for malignancy excluding NMSC was 0.8 (95% CI 0.7–1.0) using SEER as the comparator (56). These trial-program data are reassuring in absolute terms, but they were generated in selected trial populations and do not directly address the risk-enriched setting of ORAL Surveillance (56).

The long-term baricitinib dataset is similarly reassuring. In the final integrated analysis of RA trials and extensions, involving 14,744 patient-years of exposure, the incidence rate for malignancy excluding NMSC was 0.6 per 100 patient-years during the first 48 weeks (95% CI 0.34–0.91) and approximately 1.0 thereafter, while the standardized incidence ratio versus SEER was 1.07 (95% CI 0.90–1.26), suggesting no clear excess over background expectations (57). No major new malignancy-specific signal emerged over time (57). These data do not prove that baricitinib is free of cancer risk in all settings, but they do indicate that prolonged exposure in trial populations has not generated the type of unequivocal excess that would by itself establish a class-wide effect.

Upadacitinib data further complicate any simplistic class interpretation. In a 2023 integrated analysis of more than 15,000 patient-years across rheumatoid arthritis, psoriatic arthritis, ankylosing spondylitis, and atopic dermatitis, observed malignancy rates ranged from 0.3 to 1.4 events per 100 patient-years across diseases, with the lowest rates in ankylosing spondylitis and atopic dermatitis (58). Rates of NMSC (0–0.8 per 100 patient-years) and malignancy were numerically higher in the RA and PsA populations than in AS, which likely reflects both disease context and background risk (59). A broader 2025 descriptive safety analysis over more than 27,000 patient-years across IMIDs reported no major new safety signal for malignancy, though absolute malignancy rates varied by indication and remained highest in RA, the population with the oldest age and greatest comorbidity burden (58, 59). Thus, while upadacitinib long-term data do not eliminate concern, they do suggest that malignancy outcomes are strongly modified by indication and baseline patient profile, and they do not support an identical risk narrative across all diseases treated with JAK inhibition (58, 59).

### 4.4 Clinical interpretation

The most defensible interpretation of the JAK inhibitor literature is not that all agents uniformly increase cancer risk in all IMIA populations, but that a clinically meaningful signal has been demonstrated for tofacitinib in risk-enriched RA, and that this signal is strongest relative to TNF inhibitors rather than to placebo or methotrexate (8, 9, 49). Outside that setting, the evidence is more heterogeneous. Real-world studies either show no significant increase (STAR-RA), a modest non-significant increase (U.S. claims), or a small but statistically significant excess emerging with longer exposure or in selected high-risk subgroups (RABBIT) (24, 51, 53). At the same time, the signal for NMSC is increasingly difficult to dismiss, having now appeared in randomized comparisons, integrated analyses, and national registry data (49, 52, 58).

**Figure 2:**
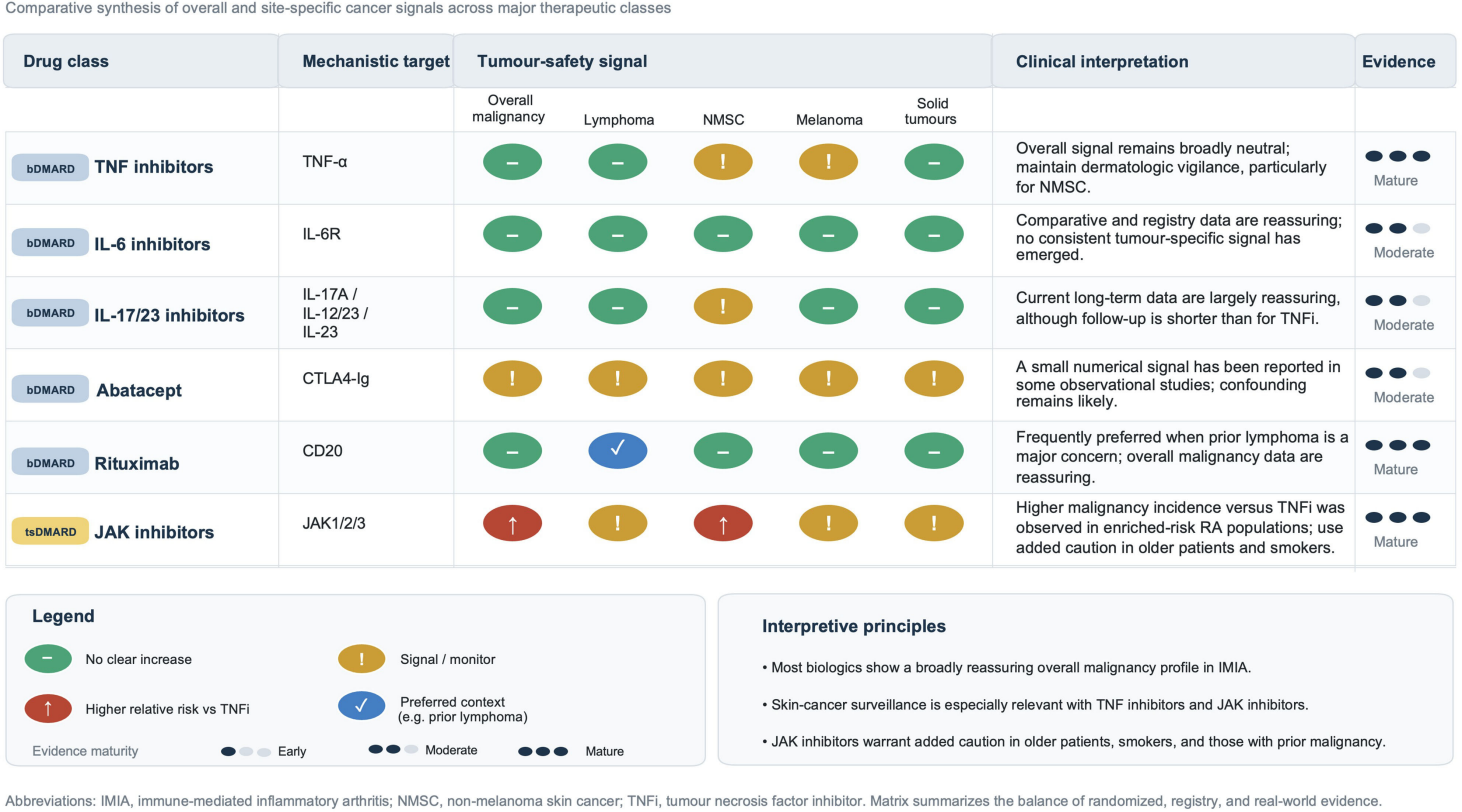
Malignancy safety profile of biologic and targeted therapies in IMIA. This evidence matrix summarizes current data regarding overall and site-specific malignancy risk associated with biologic and targeted synthetic disease-modifying antirheumatic drugs used in IMIA. Most biologic agents, including TNF inhibitors, IL-6 inhibitors and IL-17/23 inhibitors, show broadly reassuring overall malignancy profiles in large observational cohorts and registry studies. Certain site-specific signals require vigilance, particularly non-melanoma skin cancer with TNF inhibitors and Janus kinase inhibitors (JAKi). Rituximab is often favored when prior lymphoma is a major concern, whereas abatacept has shown small numerical malignancy signals in some observational analyses. Randomized and real-world evidence suggests a higher malignancy incidence with JAK inhibitors compared with TNF inhibitors in enriched-risk populations, particularly older patients and smokers. Overall, available evidence supports careful patient selection and continued surveillance rather than broad restriction of biologic therapy. IMIA, immune-mediated inflammatory arthritis; NMSC, non-melanoma skin cancer; TNFi, tumor necrosis factor inhibitor.

From a practical standpoint, this means that JAK inhibitors should be contextualized rather than reflexively avoided. For younger IMIA patients without major cancer risk factors, especially outside RA, current data do not suggest a dramatic absolute malignancy burden. However, in older patients, ever-smokers, those with high cumulative inflammatory burden, multiple prior DMARD failures, or prior malignancy, the balance shifts, and the threshold for choosing a TNF inhibitor or another biologic first becomes lower (48, 50, 52, 53). The 2025 systematic review informing the updated international consensus on JAK inhibitor safety emphasized exactly this point: safety profiles across compounds and indications are broadly consistent, but the impact of age, smoking, dose, background therapy, and comorbidities meaningfully modifies risk (48). In that sense, the JAK inhibitor story reinforces a broader principle of IMIA therapeutics: cancer risk is rarely drug-only, and almost never context-free. This becomes even clearer when risk is examined from the perspective of specific tumor types, which is the focus of the next section (48). An evidence matrix summarizing overall and site-specific malignancy risks across biologic and targeted synthetic therapies used in IMIA is presented in **Figure 2**.

## 5 Associations with specific tumor types

Examining malignancy through the lens of specific tumor types is more informative than relying on pooled “all cancer” endpoints alone. Overall malignancy analyses can dilute clinically important site-specific signals, especially when the underlying biology differs across tumor types and when event numbers are low. In IMIA, the most relevant malignancy patterns concern lymphoma, non-melanoma skin cancer (NMSC), melanoma, and lung cancer, whereas associations with most other solid tumors remain weaker, less consistent, or strongly confounded by baseline disease risk and host factors (6, 7, 24, 48).

### 5.1 Lymphomas

Lymphoma remains the classic malignancy of concern in rheumatoid arthritis because RA itself carries an increased baseline lymphoma risk, and that risk correlates strongly with cumulative inflammatory burden rather than with antirheumatic therapy alone (17). In the seminal Swedish case-control study by Baecklund and colleagues, lymphoma risk rose dramatically in the highest deciles of cumulative disease activity, strongly supporting the concept that uncontrolled inflammation is a major causal driver (17). Subsequent registry data from the British Society for Rheumatology Biologics Register did not show an overall excess of lymphoma in TNF inhibitor–treated RA compared with biologic-naive RA after adjustment for disease characteristics (7). A 2024 systematic review and meta-analysis reached a similar conclusion, finding no significant overall increase in lymphoma risk with anti-TNF therapy versus conventional therapy, although observational heterogeneity remained substantial (7, 17, 26).

That said, lymphoma risk under TNF inhibition may not be entirely uniform by subtype. In a SEER-Medicare case-control study of older Americans with RA, TNF inhibitor exposure was associated with increased odds of non-Hodgkin lymphoma overall (aOR 1.28, 95% CI 1.06–1.56), driven specifically by follicular lymphoma (aOR 2.63, 95% CI 1.63–4.24), while no increase was seen for other common NHL subtypes or for most other solid cancers (60). This does not overturn the broader reassuring registry signal, but it highlights that a “neutral” class-level interpretation may obscure subtype-specific associations in older, cancer-prone populations. Thus, for lymphoma, the most accurate conclusion is that baseline RA biology remains the dominant determinant, but some treatment-specific heterogeneity—particularly with TNF inhibitors in selected datasets—cannot be dismissed outright (7, 17, 26, 60).

For non-TNF biologics, convincing lymphoma-specific signals have not emerged. Rituximab is particularly notable because it is itself an anti-lymphoma therapy and is often favored in RA patients with prior lymphoproliferative malignancy (33, 45, 46). Abatacept has not shown a reproducible lymphoma excess, although observational datasets suggest small overall cancer differences that likely reflect confounding (42, 44). By contrast, ORAL Surveillance reported numerically more lymphomas with tofacitinib than with TNF inhibitors, contributing to the overall malignancy signal in risk-enriched RA (49). However, outside that trial, a clear class-wide lymphoma excess with JAK inhibitors has not been consistently replicated in registries, where overall cancer signals are more heterogeneous and site-specific lymphoma results remain underpowered (52, 53). Clinically, this means lymphoma risk should still be interpreted primarily through the lens of RA severity and prior lymphoproliferative history, with therapy choice acting as a modifier rather than the dominant cause.

### 5.2 Non-melanoma skin cancer and melanoma

Among all tumor categories, cutaneous malignancies are the most reproducible treatment-associated signal across advanced therapies in IMIA. For TNF inhibitors, this association is well established. In the SEER-Medicare analysis of older adults with RA, TNF inhibitor exposure was associated with increased NMSC overall (aOR 1.32, 95% CI 1.06–1.63) (60). A broader meta-analysis across inflammatory diseases found that biologic-treated patients had a significantly increased NMSC risk compared with patients with the same disease who did not receive biologics (RR 1.25, 95% CI 1.14–1.37), with a similar effect size in RA (RR 1.24) and a stronger signal emerging with treatment durations of at least two years (61). These findings fit well with the idea that biologic therapy, especially in chronically inflamed and treatment-exposed patients, may compromise cutaneous immune surveillance sufficiently to increase keratinocyte carcinogenesis.

The melanoma signal is more nuanced. In the Swedish nationwide RA cohort, TNF inhibitor exposure was associated with a modest increase in invasive melanoma risk (27). However, a large pooled collaborative analysis across 11 European biologic registers did not confirm a statistically significant overall melanoma increase with TNF inhibitors relative to biologic-naive RA (28). This discrepancy likely reflects small event numbers, different background UV exposure, and variable screening intensity rather than a fundamental contradiction. In practice, melanoma risk under TNF inhibitors should be regarded as possible but not definitively established, whereas NMSC—especially squamous cell carcinoma—has a firmer evidence base (27–29, 60, 61).

For JAK inhibitors, the cutaneous signal is even more consistent. ORAL Surveillance demonstrated higher NMSC incidence with tofacitinib than with TNF inhibitors (49), and the Swedish ARTIS study found an increased NMSC risk with JAK inhibitors versus TNF inhibitors (HR 1.39, 95% CI 1.01–1.91), which strengthened after two years of exposure (HR 2.12, 95% CI 1.15–3.89) (52). In psoriasis and psoriatic arthritis populations, a systematic review and meta-analysis reported low absolute melanoma incidence overall (0.08 per 100 patient-years) but higher NMSC incidence (0.45 per 100 patient-years), with NMSC rates being numerically higher in patients treated with JAK inhibitors than in those treated with biologics (62). These data suggest that JAK inhibition may not substantially amplify melanoma risk, but it likely does increase susceptibility to NMSC, particularly in patients with cumulative actinic damage, prior skin cancer, or prolonged treatment exposure.

Other biologics appear more reassuring, though not entirely free of concern. Abatacept has shown a small observational signal for NMSC (42, 44), while IL-17/23 inhibitors have thus far maintained low absolute melanoma and NMSC rates in long-term pooled analyses (35–41). Overall, the skin cancer evidence supports a practical hierarchy: NMSC is the most credible class-associated malignancy signal across targeted therapies, especially with TNF inhibitors, abatacept, and JAK inhibitors; melanoma remains a lower-frequency and less consistently replicated concern that still warrants vigilance in patients with personal or phenotypic risk factors.

### 5.3 Lung cancer

Lung cancer deserves separate discussion because it reflects a convergence of disease biology, smoking exposure, pulmonary comorbidity, and treatment effects. Even before therapy is considered, RA itself is associated with a higher lung cancer risk. A 2021 meta-analysis found that RA was associated with an overall lung cancer relative risk of 1.44 (95% CI 1.31–1.57), with a stronger signal in men (63). A 2024 nationwide Korean cohort confirmed this pattern, reporting an adjusted HR of 1.49 (95% CI 1.34–1.66) for lung cancer in RA versus matched non-RA controls, with greater excess among current or former heavy smokers and male patients (64). More recently, a large VA-based study demonstrated that RA was associated with a >50% increased lung cancer risk overall (aHR 1.58, 95% CI 1.52–1.64), and that RA-associated interstitial lung disease (RA-ILD) marked a particularly high-risk subgroup (aHR 3.25 for prevalent RA-ILD vs non-RA) (65). These findings are crucial because they mean that any treatment-associated lung cancer signal is superimposed on a background of already elevated risk.

Within that high-risk background, the strongest drug-associated lung cancer signal has emerged for JAK inhibitors. In ORAL Surveillance, lung cancer was the most frequently reported malignancy among tofacitinib-treated patients, and the signal was most apparent with the 10 mg twice-daily dose (49). The 2025 older-Americans SEER-Medicare analysis extended this concern by showing that JAK inhibitor exposure was not associated with increased overall cancer risk but was associated with higher lung cancer odds (OR 1.40, 95% CI 1.06–1.87), particularly in men (OR 2.12) and in those with more than two years of exposure (54). This is one of the clearest examples in which a class may appear neutral for “overall cancer” while still carrying a meaningful site-specific signal. It also explains why lung cancer risk—and especially smoking history—has become central to the clinical conversation around JAK inhibitors.

By contrast, biologic therapies have not shown a comparably consistent lung cancer excess. TNF inhibitors were not associated with lung cancer in the older-Americans case-control study (60), and non-TNF biologics likewise have not demonstrated robust site-specific lung cancer signals in modern comparative cohorts (25, 26, 33). There may still be isolated numerical imbalances, particularly in older or highly comorbid patients, but the strongest inference from current data is that lung cancer in IMIA is driven primarily by disease-, host-, and exposure-related factors, with JAK inhibitors acting as a possible additional amplifier in susceptible populations rather than a universal cause. Clinically, this supports a low threshold for smoking cessation counseling, consideration of lung cancer screening in eligible patients, and greater caution with JAK inhibitor prescribing in smokers, patients with chronic lung disease, or those with RA-ILD.

### 5.4 Other site-specific solid tumors

Beyond skin and lung malignancies, the evidence for most site-specific solid tumors remains weak, inconsistent, or hypothesis generating. In the 2025 SEER-Medicare analysis, neither TNF inhibitors nor other biologics were associated with an overall increase in cancer risk, and most specific cancer types were likewise not associated with medication exposure (54). An inverse association between JAK inhibitor exposure and breast cancer was observed in women (OR 0.62, 95% CI 0.39–0.97), but this finding is difficult to interpret biologically and should be regarded as exploratory rather than practice changing (31). Similarly, long-term meta-analysis in psoriasis/PsA found no increase in breast or prostate cancer with TNF inhibitor therapy (32). These data argue against broad concern for common hormone-sensitive or gastrointestinal solid tumors under current biologic therapy, at least at the population level.

A more subtle issue concerns renal and urological cancer. Evidence remains weak, inconsistent, and hypothesis-generating: some long-term observational datasets have suggested low-level urinary tract cancer signals across biologic classes, but these findings are exploratory and are not clearly attributable to a single mechanism or drug class. Renal cell carcinoma is a particularly relevant example because it is an immunogenic tumor in which checkpoint blockade has become an established therapeutic strategy (22). Thus, in theory, therapies that attenuate T-cell priming or broader interferon-dependent immune surveillance could alter host–tumor equilibrium in susceptible individuals. However, a 2025 nationwide cohort found that RA itself was associated with increased kidney cancer risk (aHR 1.34, 95% CI 1.04–1.78), whereas bladder and prostate cancer were not clearly increased (66). This makes it difficult to determine whether occasional urinary tract signals seen in drug-exposed cohorts reflect therapy effects, disease biology, surveillance differences, or residual confounding. At present, there is insufficient evidence to support class-specific screening strategies for urinary tract malignancy beyond usual clinical vigilance.

### 5.5 Synthesis across tumor types

Viewed together, the tumor-type evidence supports three practical conclusions. First, lymphoma, NMSC, and lung cancer are the malignancies most tightly linked to the intersection of IMIA biology and targeted therapy exposure. Second, NMSC is the most reproducible treatment-associated signal across classes, while lung cancer is the clearest site-specific concern for JAK inhibitors in high-risk RA. Third, for most other solid tumors, currently available evidence remains either reassuring or too inconsistent to justify therapy class–specific alarm. This means that malignancy counseling in IMIA should be neither generic nor drug-centric alone. It should instead be tumor-specific and risk-stratified, integrating disease severity, smoking history, prior skin cancer, pulmonary comorbidity, previous malignancy, and the particular safety profile of the selected therapy (24, 48, 54, 60–66).

## 6 Factors influencing tumor risk in treated IMIA patients

Tumor risk in IMIA cannot be attributed to drug exposure alone. Rather, it reflects the interaction of disease-related, patient-related, and treatment-related determinants that shift the balance between inflammation-driven oncogenesis and antitumor immune surveillance. This is why apparently similar therapies can have different safety profiles across studies, and why the same drug may be acceptable in one patient but less suitable in another. Contemporary guidance increasingly emphasizes individualized risk assessment, particularly in older patients, smokers, and cancer survivors, rather than generalized therapeutic avoidance (10, 15, 48).

### 6.1 Disease-related factors: inflammatory burden, disease duration, and disease phenotype

Among disease-related determinants, persistent inflammatory activity remains one of the most important. In RA, cumulative inflammatory burden is strongly associated with lymphoma development, and this relationship is especially pronounced for diffuse large B-cell lymphoma, the dominant lymphoma subtype in RA-associated lymphomagenesis (17, 67). These observations are clinically important because they imply that inadequate disease control may itself be oncogenic, particularly for hematologic malignancy. This also helps explain why some targeted therapies can appear oncologically neutral despite their immunomodulatory effects: the reduction in inflammation-driven risk may offset at least part of the risk attributable to impaired immune surveillance (7, 17, 67).

Disease phenotype also matters. RA has a more robust background association with lymphoma and lung cancer than PsA or axSpA, which means that malignancy findings derived from RA cohorts should not be transferred wholesale to other IMIA populations. Pulmonary phenotype is particularly relevant. RA-associated interstitial lung disease (RA-ILD) identifies a subgroup with markedly increased lung cancer susceptibility, and this likely reflects a combination of chronic epithelial injury, smoking enrichment, and inflammatory remodeling rather than therapy alone (65, 68). Likewise, seropositive RA appears to carry higher long-term lung cancer risk than seronegative disease, even after adjustment for smoking in some population-based analyses (68). Thus, the same treatment may operate against a very different background oncologic terrain depending on disease subtype, serologic status, and organ involvement.

Disease duration and refractory disease course likely add to this burden. In the German RABBIT register, a stronger excess malignancy signal with JAK inhibitors was seen in patients with high disease activity and multiple prior csDMARD failures, which may indicate that refractory inflammation and repeated treatment exposure are themselves markers of higher intrinsic oncologic risk (53). Similar reasoning likely applies more broadly across biologic-treated populations: patients who reach later-line therapy often have a more treatment-resistant, inflammatory, comorbidity-rich disease trajectory, and this can confound drug-specific cancer comparisons if not interpreted carefully (24, 25, 53).

### 6.2 Patient-related factors: age, smoking, prior malignancy, and cutaneous susceptibility

Age is one of the strongest modifiers of observed malignancy risk. It raises baseline cancer incidence independent of IMIA and appears to amplify treatment-associated signals in several datasets. In ORAL Surveillance, the subgroup most clearly enriched for excess malignancy risk with tofacitinib versus TNF inhibitors was defined in large part by age ≥65 years and/or smoking history (50). In RABBIT, the relative increase in malignancy observed with JAK inhibitors versus bDMARDs was more pronounced in patients aged ≥60 years (53). Age therefore acts not only as a background risk factor, but also as a practical threshold that changes how clinicians should interpret therapy-associated risk.

Smoking is equally important, especially because it links multiple relevant outcomes: RA severity, lung cancer, chronic lung disease, and possibly impaired immune surveillance. In RA cohorts, smoking consistently magnifies lung cancer risk; in a Swedish population-based study, RA patients who were ever-smokers had almost seven times the lung cancer risk of never-smoking population controls, and seropositivity further amplified that risk (68). A Korean nationwide cohort similarly found the RA-associated increase in lung cancer to be most prominent among current or former heavy smokers (64). Smoking history also contributes to the risk-enriched profile in which JAK inhibitors appear least favorable (50). These data make smoking one of the most actionable risk modifiers in clinical practice: it should influence both therapeutic selection and screening vigilance.

A history of prior malignancy is another major determinant, but its impact is highly dependent on cancer type, time since remission, and the intended therapy. The 2024 EULAR points to consider emphasize that recurrence risk should be assessed individually, in collaboration with oncology, rather than treated as a binary contraindication to targeted therapy (10). The accompanying systematic review found no overall increase in new or recurrent cancer with targeted therapies versus csDMARDs in patients with prior malignancy (15). A 2025 meta-analysis focused specifically on biologics reached a similar conclusion, reporting no clear increase in new or recurrent cancer for TNF inhibitors, IL-12/23 inhibitors, or vedolizumab compared with conventional systemic therapy or no therapy in IMIDs with previous cancer (69). This does not mean that all therapies are interchangeable after cancer, but it does mean that prior malignancy should be viewed as a risk-stratification variable, not an automatic reason to withhold effective therapy.

Cutaneous susceptibility is another underappreciated patient-level factor. Fair skin, cumulative UV exposure, prior NMSC, extensive actinic damage, and in psoriatic disease, prior PUVA or other phototherapy exposures can substantially alter the clinical relevance of drug-associated skin cancer signals. This is particularly important because the most reproducible malignancy signal across advanced IMIA therapies is cutaneous, especially for NMSC. In a 2025 active-comparator study of inflammatory diseases, the increase in cutaneous malignancy was driven predominantly by TNF inhibitor exposure, whereas no other biologic class showed a comparably strong independent signal (70). Meanwhile, melanoma meta-analytic data remain inconclusive: biologic-treated patients did not show a statistically significant melanoma increase, but clinically meaningful increases could not be ruled out because available studies were underadjusted for key host factors (71). In practice, this means that patients with prior skin cancer or strong cutaneous risk factors deserve a different counseling and monitoring strategy than those without such background susceptibility (61, 70, 71).

### 6.3 Treatment-related factors: latency, cumulative exposure, line of therapy, and immunosuppressive burden

The effect of treatment duration on malignancy risk is one of the clearest signals emerging from recent literature. In ORAL Surveillance, the malignancy curves for tofacitinib and TNF inhibitors separated only after approximately 18 months, suggesting a latency-sensitive effect (49). In Swedish ARTIS data, the NMSC signal with JAK inhibitors strengthened after at least two years of exposure (52). In RABBIT, a relative increase in malignancy was evident primarily in treatment episodes lasting longer than 16 months (53). These convergent findings imply that short observational windows may underestimate risk, particularly for skin cancer and perhaps for some solid tumors. Thus, cumulative exposure duration is not merely a methodological nuisance; it is biologically and clinically relevant.

Line of therapy and cumulative immunosuppressive burden are closely related and frequently confounded with one another. Patients receiving later-line biologics, rituximab, abatacept, or JAK inhibitors often have more severe disease, more prior csDMARD exposure, longer disease duration, and greater comorbidity than TNF inhibitor initiators. This pattern can create apparent malignancy differences that may reflect patient selection as much as pharmacology. The modestly higher cancer rates seen with abatacept, rituximab, or JAK inhibitors in some real-world studies are a good example (24, 25, 42–44). Likewise, the stronger JAK inhibitor signal in RABBIT among patients with ≥3 prior csDMARDs suggests that cumulative treatment history may partly function as a surrogate for refractory disease and total immunologic injury rather than acting as a simple “dose count” (53). The practical implication is that treatment comparisons must be interpreted in the context of who receives what, and when.

A related issue is background therapy, including concomitant glucocorticoids or methotrexate. Although these drugs are difficult to isolate as independent malignancy determinants in observational cohorts, they likely contribute to the overall immunosuppressive burden and may also act as markers of incomplete disease control. Recent JAK inhibitor safety reviews have therefore argued that risk assessment should not be drug-centric alone, but should account for total therapeutic exposure, steroid dependence, chronic lung disease, smoking, and age as part of a unified vulnerability profile (48). In other words, malignancy risk under advanced therapy is best understood not as the property of a single molecule, but as the emergent result of a patient’s cumulative inflammatory and immunomodulatory history (24, 48, 53).

### 6.4 Overall interpretation

Taken together, the factors influencing tumor risk in treated IMIA patients are not additive in a simple linear way. High inflammatory burden, older age, smoking exposure, prior malignancy, skin cancer susceptibility, prolonged therapy duration, and heavy prior treatment history all interact to shape the observed safety profile of a given agent. This is why malignancy counseling cannot be reduced to “drug A is safer than drug B.” Instead, it requires matching the treatment to the patient’s baseline oncologic terrain. A younger PsA patient without smoking history or prior cancer is fundamentally different from an older seropositive RA patient with RA-ILD, prior NMSC, and multiple prior DMARD failures. Contemporary EULAR guidance and modern safety reviews both converge on this same principle: malignancy risk in IMIA should be individualized, phenotype-aware, and dynamically reassessed over time (10, 15, 48, 69).

## 7 Safety management strategies and clinical recommendations

The clinical management of malignancy risk in IMIA should not be reduced to a binary choice between “safe” and “unsafe” drugs. Instead, it requires a structured approach that integrates baseline oncologic risk, disease severity, treatment alternatives, and the strength of the available evidence for each drug class. Recent guidance has moved decisively away from blanket avoidance of targeted therapy in cancer-exposed patients and toward individualized, multidisciplinary, risk-adapted prescribing (10, 15, 48).

### 7.1 Baseline oncologic risk assessment before advanced therapy

Before initiating or switching to a biologic or targeted synthetic DMARD, clinicians should perform a focused malignancy-oriented assessment. At minimum, this should include prior cancer history (type, stage, treatment, and remission status), smoking exposure, chronic lung disease or RA-associated interstitial lung disease, prior non-melanoma skin cancer, cumulative ultraviolet damage, family history of malignancy, and previous immunosuppressive burden. This baseline appraisal is especially important because many of the strongest treatment-associated signals—such as lung cancer with JAK inhibitors or NMSC with TNF inhibitors and some other agents—become clinically meaningful only in patients who are already risk-enriched (10, 15, 48, 49, 52).

Risk assessment should also include verification that the patient is up to date with routine cancer screening according to general-population recommendations. For example, the current U.S. Preventive Services Task Force (USPSTF) recommends biennial breast cancer screening from age 40 to 74, colorectal cancer screening from age 45 to 75 (with selective screening from 76 to 85), and annual low-dose CT for lung cancer screening in adults aged 50 to 80 years with a 20 pack-year smoking history who currently smoke or quit within the past 15 years (72–75). These are not IMIA-specific protocols, but adherence becomes especially important in patients receiving long-term immunomodulatory therapy. In this sense, “cancer safety management” begins not with the drug choice itself, but with bringing standard preventive care fully up to date before treatment starts.

### 7.2 Choosing therapy in patients with previous malignancy

The most important recent advance in this area is the 2024 EULAR points to consider for initiating targeted therapy in patients with inflammatory arthritis and a history of cancer (10). The central message is that therapy should be based on individualized recurrence risk rather than reflexive avoidance of all targeted drugs. When the previous malignancy is in remission, appropriate targeted therapy should not be unnecessarily delayed, because undertreated inflammatory arthritis has its own consequences for function, comorbidity, and potentially malignancy risk (10, 15).

Within this framework, treatment selection should be guided by prior tumor type, time since remission, host risk factors, and oncology input. TNF inhibitors may be preferred when targeted therapy is indicated in patients with previous solid cancer in remission, whereas B-cell depletion with rituximab may be favored in those with prior lymphoma. By contrast, JAK inhibitors and abatacept should be used with caution and preferably only when suitable alternatives are lacking, reflecting both limited survivorship-specific evidence and unresolved safety signals in selected populations (10).

Supporting this approach, the accompanying EULAR systematic review found no overall increase in new or recurrent cancer with targeted therapies compared with csDMARDs in patients with prior malignancy, and a 2025 meta-analysis of biologics in immune-mediated inflammatory disease reached a similar conclusion (15, 69). A nationwide Danish register-based cohort of 720 RA patients with prior solid tumors in remission similarly found no increased cancer recurrence with biologic DMARD therapy compared with conventional DMARD therapy (HR 0.92, 95% CI 0.38–1.73) (76). These data support individualized, oncology-coordinated treatment rather than prolonged undertreatment based on cancer history alone. A recent safety systematic literature review informing the 2025 EULAR RA recommendations similarly underscored that non-melanoma skin cancer risk should be monitored, while available data do not consistently attribute this risk to a single DMARD class (77).

### 7.3 Incorporating JAK inhibitor safety warnings into daily practice

JAK inhibitors require a more formal risk-mitigation process than most biologics. Following ORAL Surveillance, the U.S. FDA required boxed warnings for tofacitinib, baricitinib, and upadacitinib regarding serious cardiovascular events, cancer, thrombosis, and death, and limited their use in chronic inflammatory conditions to patients who have not responded to or cannot tolerate one or more TNF blockers (78). The FDA specifically highlighted higher rates of lymphoma and lung cancer with tofacitinib than with TNF blockers in RA and emphasized current or past smoking and known malignancy as important risk modifiers (78). The European Medicines Agency subsequently adopted a similar but even more explicitly risk-stratified position, recommending that JAK inhibitors be used only if no suitable alternatives are available in patients aged ≥65 years, in current or past long-term smokers, in those at increased cardiovascular risk, and in those at increased cancer risk (79).

In practical terms, this means that JAK inhibitor prescribing should include a documented benefit–risk conversation rather than routine escalation. The discussion should address age, smoking history, previous malignancy (other than successfully treated NMSC), lung disease, skin cancer history, and alternative biologic options (48, 78, 79). If a JAK inhibitor is selected, clinicians should avoid unnecessary concomitant immunosuppressive burden where possible, reinforce adherence to screening, and re-evaluate the treatment strategy over time—particularly beyond the first 18–24 months, when some datasets suggest risk curves begin to diverge (49, 52, 53). The goal is not to prohibit JAK inhibitors universally, but to ensure they are used in patients whose overall profile makes the expected therapeutic benefit proportionate to the observed safety signal.

### 7.4 Skin cancer prevention and surveillance

Because NMSC is the most reproducible treatment-associated malignancy signal across advanced therapies, cutaneous risk management deserves dedicated attention. Available evidence supports heightened vigilance in patients receiving TNF inhibitors, JAK inhibitors, and possibly abatacept, especially when additional host factors are present. These include prior NMSC, fair skin, extensive actinic damage, prior PUVA or other high cumulative ultraviolet exposure, and prolonged immunomodulatory therapy (31, 43, 52, 61, 70). While there is no universal rheumatology guideline mandating dermatologic examination intervals for all biologic users, periodic full-skin examination is a reasonable strategy in high-risk patients, and this approach is consistent with both the pattern of evidence and the precautionary logic reflected in abatacept-associated NMSC analyses (43, 70).

Patient education is equally important. Clinicians should encourage photoprotection, prompt reporting of non-healing or changing skin lesions, and a low threshold for dermatology referral after suspicious findings. This is especially relevant when the therapy-associated risk is not a large increase in fatal cancer, but rather a modest, cumulative increase in treatable keratinocyte carcinomas. A preventive strategy based on awareness and early detection is therefore more appropriate than wholesale treatment avoidance in most patients (43, 61, 70).

### 7.5 Management when malignancy develops during therapy

Evidence on how to manage incident malignancy arising during biologic or targeted therapy remains limited, and high-quality comparative data on stopping versus continuing treatment are lacking. Accordingly, most recommendations rely on expert consensus and multidisciplinary practice rather than formal trials. A reasonable general approach is to pause the immunomodulatory agent at the time of cancer diagnosis, involve oncology promptly, and reassess the arthritis treatment plan once the cancer type, stage, prognosis, and immediate treatment strategy are defined (10, 15). This approach is particularly important for hematologic malignancies, melanoma, and cancers requiring intensive systemic therapy, where immunologic interference or infection risk may be especially relevant.

After malignancy treatment, decisions regarding re-initiation should again be individualized. If the cancer is in remission and inflammatory disease activity requires renewed targeted therapy, EULAR supports reintroducing an appropriate agent without unnecessary delay, using the same risk-stratified framework applied at baseline (10). In practice, this often means favoring TNF inhibitors for selected prior solid cancers, rituximab after lymphoma, and reserving JAK inhibitors or abatacept for situations in which alternatives are unsuitable or ineffective (10, 15). The core principle is continuity of disease control without abandoning oncologic prudence.

### 7.6 Shared decision-making and a practical framework

The final step in malignancy risk management is to make the process explicitly shared. Patients should understand that the risk associated with advanced therapy is not uniform, that untreated disease also carries meaningful risks, and that their personal characteristics—age, smoking, prior cancer, actinic damage, and pulmonary comorbidity—meaningfully influence treatment choice (10, 48, 78, 79). In practical terms, a usable framework can be summarized as follows:

1. Estimate baseline cancer risk before choosing a therapy.
2. Update standard cancer screening before or soon after treatment initiation.
3. Prefer therapies with the most reassuring evidence when prior cancer or strong host risk factors are present.
4. Use JAK inhibitors more selectively, especially in older patients, smokers, and those with known malignancy risk.
5. Intensify skin surveillance when NMSC risk factors are present.
6. Coordinate with oncology early in cancer survivors and whenever new malignancy develops.

Reassess dynamically, because risk evolves with age, duration of exposure, and changing comorbidity burden. **Table 2** provides a risk-stratified clinical framework for therapy selection and malignancy surveillance in IMIA.

**Table 2:** Risk-stratified clinical framework for therapy selection and malignancy surveillance in IMIA.

Clinical context	Preferred approach	Monitoring priorities
Prior solid cancer in remission	Consider TNF inhibitor or phenotype-matched non-TNF biologic after oncology input; avoid JAK inhibitors or abatacept when suitable alternatives exist.	Standard cancer surveillance and shared decision-making with oncology.
Prior lymphoma	Consider rituximab-based strategy when clinically appropriate; avoid JAK inhibitors unless the indication is compelling.	Hematology/oncology coordination; monitor lymphadenopathy and B symptoms.
Older age or major smoking history	Prefer agents with reassuring long-term comparative safety; use JAK inhibitors only after documented benefit-risk discussion.	Smoking cessation, lung cancer screening eligibility, and periodic review of treatment necessity.
High skin cancer susceptibility or prior NMSC	Any effective agent may be used with surveillance; consider lower-cutaneous-risk alternatives when feasible.	Regular skin examination, photoprotection, and low threshold for dermatology referral.
Incident malignancy during therapy	Temporarily pause or individualize therapy pending oncologic staging and treatment plan; avoid automatic re-challenge.	Oncology-led coordination; re-initiation based on remission status and arthritis severity.

This table provides a simplified risk-stratified framework linking common clinical contexts with preferred treatment approaches and monitoring priorities in patients with IMIA receiving biologic or targeted synthetic DMARDs.

IMIA, immune-mediated inflammatory arthritis; TNFi, tumor necrosis factor inhibitor; JAKi, Janus kinase inhibitor; b/tsDMARDs, biologic or targeted synthetic disease-modifying antirheumatic drugs; NMSC, non-melanoma skin cancer; CV, cardiovascular.

**Figure 3:**
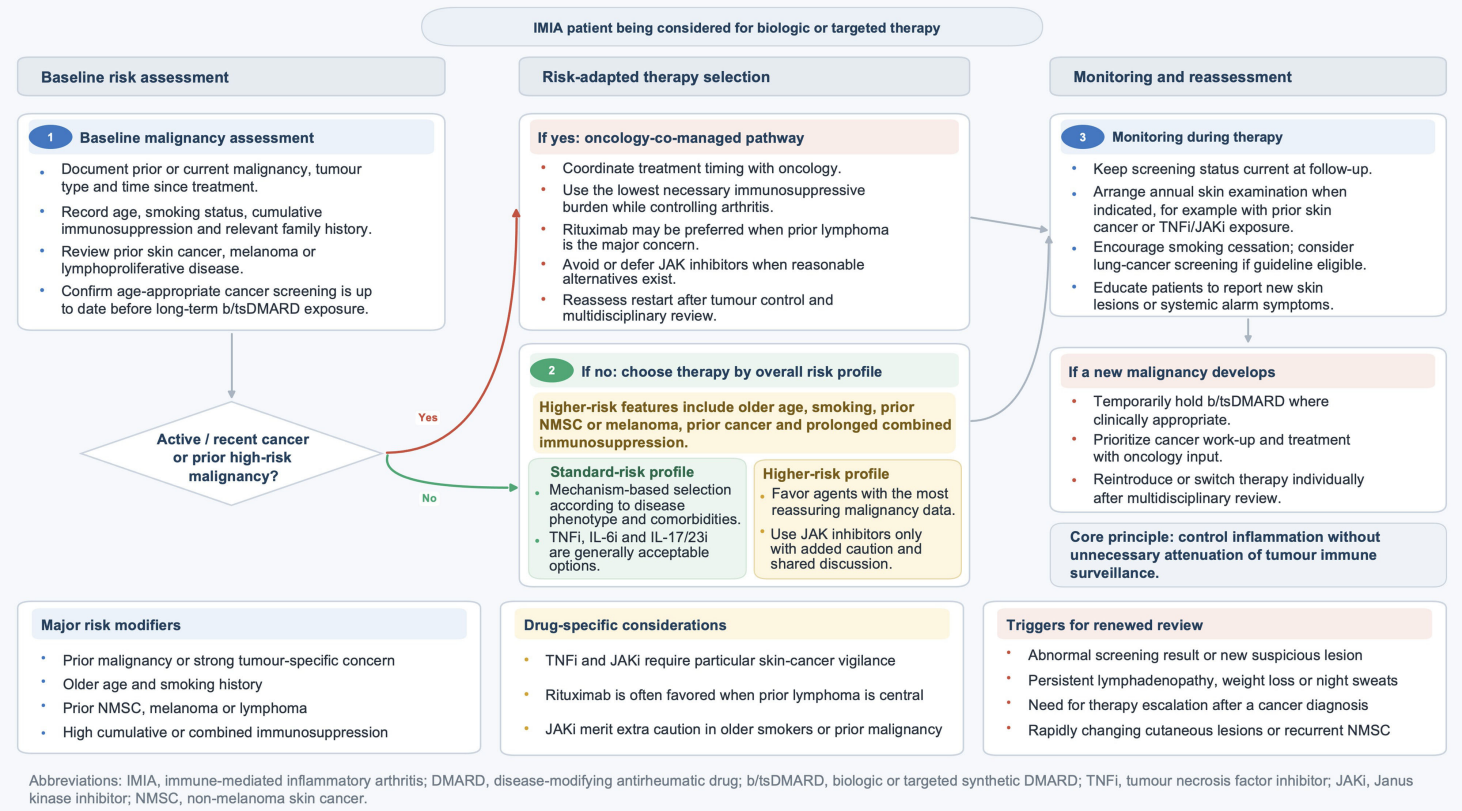
Risk-adapted management of malignancy in IMIA. Patients with IMIA who are considered for biologic or targeted synthetic disease-modifying antirheumatic drugs (b/tsDMARDs) should undergo structured malignancy risk assessment followed by risk-adapted therapy selection and ongoing monitoring. Baseline evaluation includes documentation of prior or current malignancy, tumor type and time since treatment, together with key modifiers such as age, smoking status, cumulative immunosuppression and prior skin cancer, melanoma or lymphoma. Patients with active or recent malignancy, or a history of high-risk cancer, should follow an oncology-co-managed pathway that prioritizes tumor control and cautious immunomodulatory therapy selection. In patients without high-risk cancer history, treatment may be chosen according to disease phenotype and overall risk profile, favoring agents with reassuring malignancy safety data in higher-risk individuals. Regular surveillance and multidisciplinary reassessment are essential when new malignancy is suspected or diagnosed.

This approach keeps the focus on individualized care rather than fear-driven undertreatment. In contemporary IMIA management, the most defensible strategy is not therapeutic nihilism, but risk-stratified precision prescribing grounded in disease control, oncologic context, and transparent clinician–patient decision-making (10, 15, 48). **Figure 3** outlines a practical risk-adapted management algorithm for patients with IMIA receiving biologic or targeted therapies.

### 7.7 Limitations

Several limitations should temper interpretation of this review and of the underlying evidence base. First, this article is a narrative synthesis rather than a formal systematic review or meta-analysis, and therefore it does not provide pooled estimates across all drug classes. Second, many malignancy outcomes are rare, so analyses of individual tumor types are often underpowered and may yield wide confidence intervals or unstable estimates. Third, observational studies are vulnerable to channeling bias, confounding by indication, differential cancer screening, latency effects, and incomplete capture of cumulative immunosuppressive exposure. Fourth, the evidence base is strongest for RA and TNF inhibitors, whereas comparative data in PsA, axSpA, JIA, and newer biologic classes remain less mature. Finally, evolving oncology practice, including broader use of immune checkpoint inhibitors, may change the clinical relevance of antitumor immune surveillance over time. The conclusions of this review should therefore be interpreted as a risk-stratified clinical framework rather than a definitive ranking of drug carcinogenicity.

## 8 Conclusion

Taken together, current evidence supports a risk-stratified view of tumor safety in IMIA. Most biologic therapies appear broadly reassuring with respect to overall malignancy, whereas JAK inhibitors warrant greater caution in selected high-risk populations, particularly older patients, smokers, and those with prior malignancy or prolonged treatment exposure. The central clinical challenge is therefore not therapeutic avoidance, but individualized balancing of inflammatory control against oncologic vulnerability. Future work should prioritize long-term comparative studies, site-specific cancer outcomes, and survivorship-focused treatment frameworks. Such an approach will better align precision immunotherapy with durable disease control, patient safety, and translational relevance in contemporary immunology.
